# Analysis of One-Dimensional Ivshin–Pence Shape Memory Alloy Constitutive Model for Sensitivity and Uncertainty

**DOI:** 10.3390/ma13061482

**Published:** 2020-03-24

**Authors:** A B M Rezaul Islam, Ernur Karadoğan

**Affiliations:** Robotics & Haptics Lab, School of Engineering & Technology, Central Michigan University, Mount Pleasant, MI 48859, USA; islam2a@cmich.edu

**Keywords:** shape memory alloy, Ivshin Model, Pence Model, sensitivity analysis, uncertainty analysis, SMA, shape memory alloy constitutive model, SMA model, shape memory alloy behavior

## Abstract

Shape memory alloys (SMAs) are classified as smart materials due to their capacity to display shape memory effect and pseudoelasticity with changing temperature and loading conditions. The thermomechanical behavior of SMAs has been simulated by several constitutive models that adopted microscopic thermodynamic or macroscopic phenomenological approaches. The Ivshin–Pence model is one of the most popular SMA macroscopic phenomenological constitutive models. The construction of the model requires involvement of parameters that possess inherent uncertainty. Under varying operating temperatures and loading conditions, the uncertainty in these parameters propagates and, therefore, affects the predictive power of the model. The propagation of uncertainty while using this model in real-life applications can result in performance discrepancies or failure at extreme conditions. In this study, we employed a probabilistic approach to perform the sensitivity and uncertainty analysis of the Ivshin–Pence model. Sobol and extended Fourier Amplitude Sensitivity Testing (eFAST) methods were used to perform the sensitivity analysis for simulated isothermal loading/unloading at various operating temperatures. It is evident that the model’s prediction of the SMA stress–strain curves varies due to the change in operating temperature and loading condition. The average and stress-dependent sensitivity indices present the most influential parameters at several temperatures.

## 1. Introduction

Shape memory alloys (SMAs) consist of a family of smart materials which can sustain large plastic strains that can be completely recovered with the application of heat. This behavior is known as the “shape memory effect” (SME). They also exhibit elastic response to the stress applied above a characteristic temperature forming a hysteresis loop, which is known as “Pseudoelasticity” (PE) or “Superelasticity (SE)”. These fundamental characteristics of SMAs exist due to reversible thermoelastic crystalline phase transformation between austenite phase (high temperature and low stress) and martensite phase (low temperature and high stress) as a function of stress and temperature. Both SME and PE depend on the stress–strain behavior and have been used in numerous research projects, engineering designs, and applications in medical, automobile, aerospace, industries, robotics, and consumer products.

Historically, most SMA-related applications have been in the medical fields. Some examples include, a 35-DOF (Degree of Freedom), teleoperated, SMA actuated snake robot [1] that was developed for minimally invasive surgery (MIS) of throat. Here DOF refers to configuration of a mechanical system in terms of modes in which the system can move. In robotics, this term is used to define a robots’ capability of motion. For the same MIS application, Hornblower [2] devised and tested a 4-DOF lightweight microbot. Mineta [3] developed an active catheter made of SMA actuator with helical biasing coil and covered with thick silicon rubber. Carrozza et al. [4] proposed an SMA actuator-based miniature pressure regulator which was incorporated in a miniature protype robot for performing colonoscopy. A self-propelling inchworm robot was developed by Peirs et al. [5] with a 3-DOF manipulator for colonoscopy. Morra et al. proposed a miniature gripper actuated by SMA springs and wires for laparoscopic operations [6]. Büttgenbach et al [7] proposed a mechanical micro gripper and SMA fabricated micro valve and artificial muscle actuator. Several monolithic SMA grippers were developed by Kohl et al. [8,9]. SMA wire actuated implantable drug delivery systems were developed for the treatment of cancer with chemotherapy and hormonal treatment [10]. Haga et al. developed SMA coil actuators for the treatment of intestinal obstruction [11]. In orthodontics, the first dental braces were made from a nickel and titanium-based alloy (NiTi) exploiting the pseudoelastic property of the alloy [12,13]. NiTi SMA wires have been used for years in fixed orthodontic treatment with multibrackets [14]. Pseudoelastic behavior is also being exploited for producing orthodontic distractors [15]. These distractors solve the problem of teeth overcrowding in the mandible district. In the endodontic field, which deals with the problems related with the tooth pulp and surrounding tissues, there was a necessity of rotating devices known as files for performing perfect cleaning during root canal procedure. The earlier devices were made of steel and being used manually. At the end of the 1980s, significant improvement in the procedure was made possible by the introduction of NiTi [16,17]. Due to the pseudoelastic behavior, it was assured that the NiTi files had flexibility, recovery of deformation and limitation of applied force which allowed them to be used with rotating motor [18]. In the orthopedic field, fracture treatment is done by orthopedic staples where SMA generated stress is exploited to join two fractured pieces due to heating in constrained environment [19]. In [20], a NiTi plate was used for the treatment of mandible fracture. NiTi rods are also inserted in devices for treating scoliosis [21,22,23] where vertebrae relative position is modified by constrained recovery. SMAs are also being used in the vascular field in biomedical applications [24,25,26,27].

In automotive applications, many manufacturers have been actively implementing SMAs to their vehicles to perform various functions. An example of relatively early application of SMA is an actuator that was used as a thermally responsive pressure control valve by Mercedes–Benz for smooth gear-shifting [28], which was introduced in 1989. Later, Alfmeier Präzision, AG successfully completed the mass production of SMA pneumatic valves for supporting lumbar region of passengers in car seats for Daimler Mercedes–Benz [29], which is currently used by most major automotive manufacturers in their production vehicles. General Motors have obtained more than 200 patents in their endeavors with SMAs since mid-1990s. The first GM vehicle was seventh generation Chevrolet Corvette with SMA actuator in which SMA was used to actuate the hatch vent for closing the trunk lid easily [30]. Centro Ricerche Fiat (CRF) has developed numerous patented applications of SMA for devices such as electrically actuated antiglare rear-view mirror, headlamp actuators, fuel filling lid actuator, and locking mechanism [31,32,33,34].

In aerospace applications, lifting body performance optimization was done for a Smart Wing program using active materials that include SMAs [35,36,37,38]. A research project was performed that used bending actuation of SMAs. The objective was to trade-off between mitigating noise at take-off and landing and performance at altitude [39,40,41,42]. Also, there has been significant research to apply SMAs in the main rotor of the aircraft [43]. In an earlier study, SMA torque tubes were used for varying the twist of rotor blade on a tiltrotor aircraft [44]. In that study, onboard actuation was provided by the shape recovery of the torque tube which allowed significantly different blade configuration requiring for optimization of the tiltrotor performance.

In other applications, superelasticity of NiTi powder is being used for enhancement of the resistance of SnPbAg solder to thermal fatigue [45]. Cracks and ruptures in solders for joining electronic devices are often formed in printed circuit boards (PCB) when subjected to significant stress. Cu-coated NiTi powder enforced SnPbAg solder shows improved stiffness and ductility without degrading the electrical conductivity. For domestic safety, SMA thermal actuators are developed. In household and hospitality buildings such as hotels, most frequent injuries occur due to hot water in sink, tub, and shower. A small NiTiCu element is placed in an antiscald valve which when heated to a certain temperature, close the valve. The valve reopens when the water temperature is safe [46]. In robotics field, SMAs are being used as actuators [47,48,49]. For instance, Huang et al. [50] proposed actuators which can be used as “artificial muscle” for variety of soft robotic systems which can have fast locomotion in dynamic condition. In [51], authors demonstrated a new approach in designing of a jellyfish using SMA springs as artificial muscles which imitates morphology and kinematics of an actual animal.

Along with the ubiquitous applications of SMAs, several constitutive models were developed to describe SMA behavior in terms of stress, strain, and temperature. Majority of these models are one-dimensional descriptions of the material behavior including Tanaka and Nagaki [52], Tanaka and Iwasaki [53], Tanaka, Kobayashi and Sato [54], Sato and Tanaka [55], Liang and Rogers [56], Brinson [57], Ivshin and Pence [58], Pence [59], Brinson and Lammering [60], Boyd and Lagoudas [61], and Patoor, Eberhardt and Berveiller [62,63]). Experimental data specific to a particular SMA are required to determine parameters of these models. Therefore, naturally, the model parameters are subjected to experimental uncertainty as well as random variability. Regardless of the selected constitutive model, this uncertainty in the parameters propagates to the resulting stress–strain response of the alloys after any loading-unloading or change in temperature. Thus, at different temperatures, loading and phase transformation conditions, it is necessary to be aware of which parameters affects the response of the model the most. Performing a thorough sensitivity analysis results in identifying these influential parameters. Additionally, model robustness can be tested, and input-output variable relationship can be depicted in the sensitivity analysis results. In [64], Karadogan used a probabilistic approach to analyze the Brinson SMA model sensitivity to its parameters. In that study, it was presented that how the uncertainty in the input parameters propagates to the resulting stress–strain curves. The analyses were performed at different temperature ranges and loading conditions resulting in six different cases. Islam et al. [65] performed sensitivity and uncertainty analysis of Tanaka and Liang-Rogers SMA constitutive model. In that study, both the models were analyzed at two different temperatures and loading conditions resulting in four cases to determine the parameters for which the models were most sensitive. The uncertainty analysis performed showed the uncertainty propagation of the model in terms of a variability band. The results provide useful insights in designing applications using SMAs.

As mentioned previously, the analysis of sensitivity and uncertainty for SMAs have been performed for some widely used constitutive models [64,65]. To the authors’ knowledge, however, a study to determine the influential parameters and uncertainty propagation of the Ivshin and Pence SMA constitutive model has not been performed. In this paper, the Ivshin and Pence SMA model was analyzed in terms of its sensitivity to the model parameters by means of sensitivity indices using two popular sensitivity analysis approaches. The sensitivity analysis presents the most influential parameters of the model. Additionally, to determine the propagation of uncertainty to the output stress–strain relationship due to the uncertainty present in the input parameters, uncertainty analyses have been performed. Various loading-unloading conditions and operating temperatures were simulated for the analyses.

## 2. Ivshin–Pence SMA Model

The Ivshin–Pence model is one of the most popular SMA constitutive models that describes the SMA behavior. The model is based on thermodynamic consideration using Gibbs free energy where kinetics of martensitic transformation is described by a set of thermomechanical equation. The evolution equations developed in the model govern time history of the system based on changes in stress (σ), strain (ϵ) and temperature (T).

In this model [58], the austenite fraction, α is considered to be the primary variable. Austenite fraction can be converted to martensite fraction, ξ by the following substitution:

(1) α=1−ξ

With the application of stress, the material exhibits strain. The total strain is obtained from the following equation:

(2) $$\epsilon =\left(1-\alpha \right)\epsilon_{m}+\alpha \epsilon_{a}$$

where $\epsilon_{m}$ and $\epsilon_{a}$ are the individual phase strains for martensite and austenite, respectively. These strains are defined as:

(3) $$\epsilon_{a}=\frac{\sigma }{E_{a}},\;\epsilon_{m}=\frac{\sigma }{E_{m}}+\epsilon_{L}$$

where $E_{a}$ is the elastic modulus of austenite, $E_{m}$ is the elastic modulus of martensite, and $\epsilon_{L}$ is the maximum residual strain.

Duhem–Madelung form [66] in one of the equations causes hysteresis to be inherent in the model. Duhem–Madelung type ordinary differential equations derived by Ivshin–Pence for the austenite-phase fraction describes the transformation kinetics. The differential equation of the Ivshin–Pence model when austenite transforms to martensite is:

(4) $$\frac{d\alpha }{dt}=\left[\left\{\frac{\alpha \left(t_{k}\right)}{\alpha_{max}\left(\beta (T(t_{k}\right),\sigma \left(t_{k}\right)))}\right\}\frac{d\alpha_{max}}{d\beta }\right]\left(\frac{\partial \beta }{\partial T}\frac{dT}{dt}+\frac{\partial \beta }{\partial \sigma }\frac{d\sigma }{dt}\right);\frac{d\alpha }{dt}\leq 0$$

Here, T denotes temperature. The reverse transformation, i.e., martensite to austenite transformation is modeled as:

(5) $$\frac{d\alpha }{dt}=\left[\left\{\frac{1-\alpha \left(t_{k}\right)}{1-\alpha_{min}\left(\beta (T(t_{k}\right),\sigma \left(t_{k}\right)))}\right\}\frac{d\alpha_{min}}{d\beta }\right]\left(\frac{\partial \beta }{\partial T}\frac{dT}{dt}+\frac{\partial \beta }{\partial \sigma }\frac{d\sigma }{dt}\right);\;\frac{d\alpha }{dt}\geq 0$$

In Equation (6), isofractional curves are parameterized by β which is a function of Temperature T and stress σ. Their values range from negative to positive infinity. Envelope function $\alpha_{max}$ defines one of the surfaces of hysteresis curves when austenite transforms to martensite monotonically from α=1 to α=0 and envelop function $\alpha_{min}$ indicates the other surface of hysteresis curves when martensite transforms to austenite monotonically from α=0 to α=1. $t_{k}\;\mathrm{is}$ known as “switching instants” or “transformation return points” at the last turn. β(T, σ) for the case where thermal expansion effects are neglected and austenite and martensite have constant and equal specific heats is defined as:

(6) $$\beta =T+\frac{1}{S_{ao}-S_{mo}}\left\{\frac{\left(E_{m}-E_{a}\right)}{2E_{A}E_{m}}\sigma^{2}-\epsilon_{L}\sigma \right\}$$

where $S_{ao}$ and $S_{mo}$ denote specific entropies at a reference temperature for the austenite and martensite phases, respectively. The simplest form of the envelope functions $\alpha_{max}$ and $\alpha_{min}$ are the piecewise linear functions as shown below:

(7) $$\alpha_{max}\left(\beta \right)=\{\begin{array}{l} 0;\;T\leq M_{f}\\ \frac{T-M_{f}}{M_{s}-M_{f}};\\ 1;\;M_{s}\leq T \end{array}M_{f}\leq T\leq M_{s}$$

(8) $$\alpha_{min}\left(\beta \right)=\{\begin{array}{l} 0;\;T\leq A_{s}\\ \frac{T-A_{s}}{A_{f}-A_{s}};\\ 1;\;A_{f}\leq T \end{array}A_{s}\leq T\leq A_{f}$$

Here, critical temperatures $M_{s}$, $M_{f}$, $A_{s}$ and $A_{f}$ are Martensite Start Temperature, Martensite Finish Temperature, Austenite Start Temperature and Austenite Finish Temperature respectively. To avoid various specialized technical difficulties associated with the derivative discontinuities in those functions (Ivshin [58]), the Ivshin–Pence model uses the following functions:

(9) $$\alpha_{max}\left(\beta \right)=0.5+0.5\;tanh\left(k_{1}\beta +k_{2}\right)$$

(10) $$\alpha_{min}\left(\beta \right)=0.5+0.5\;tanh\left(k_{3}\beta +k_{4}\right)$$

where $k_{1}$, $k_{2}$, $k_{3}$ and $k_{4}$ are adjustable fitting parameters. The pure martensite state α=0 and pure austenite state α=1 are achieved in the limit when β tends to negative and positive, respectively. As a result, the choice of these fitting parameters based on the critical temperatures $M_{s}$, $M_{f}$, $A_{s}$ and $A_{f}$ is open to interpretation. For example, $k_{1}$, $k_{2}$ in Equation (9) can be so chosen that the value of $\alpha_{max}\left(\beta \right)$ and its slope match as per Equation (7) at $T=\left(M_{s}+M_{f}\right)/2$ which is the intermediate temperature on the austenite-to-martensite transition path at pure stress-free state. Similar consideration can be applied to $\alpha_{min}\left(\beta \right)$ with respect to $A_{s}$ and $A_{f}$. An alternative to finding the fitting parameters is to consider a small number, Δ which represents the zero-phase fraction. Consequently, $k_{1}$, $k_{2}$, $k_{3}$ and $k_{4}$ are chosen such that $\alpha_{max}\left(M_{f},0\right)=\alpha_{min}\left(A_{s},0\right)=\Delta \;\mathrm{and}\;\alpha_{max}\left(M_{s},0\right)=\alpha_{min}\left(A_{f},0\right)=1-\Delta$ (Ivshin [58]). In Ivshin [58], this later approach with Δ = 0.02 has been considered.

Using the above envelope functions (Equations (9) and (10)), isofractional curves and integrating Equations (4) and (5), the fundamental and final constitutive equations of the model are obtained as:

(11) $$\alpha =\{\begin{array}{c} 1-\left\{\frac{1-\alpha \left(t_{k}\right)}{1-\alpha_{\min}\left(\beta (T(t_{k}\right),\sigma \left(t_{k}\right)))}\right\}\left\{1-\alpha_{\min}\left(\beta \left(T,\sigma \right)\right)\right\};\;\frac{d\alpha }{dt}\geq 0\\ \frac{\alpha \left(t_{k}\right)}{\alpha_{\max}\left(\beta (T(t_{k}\right),\sigma \left(t_{k}\right))}\alpha_{\max}\left(\beta \left(T,\sigma \right)\right);\;\frac{d\alpha }{\mathrm{dt}}\leq 0 \end{array}$$

## 3. Methods

To perform the sensitivity and uncertainty analyses of the Ivshin–Pence model, a MATLAB library was developed to simulate the material behavior as per the model. The material properties were obtained from Ivshin et al. [58] for validation purposes during the development stage of the library and are presented in Table 1. The critical temperatures for the material were $M_{s}=22$ °C, $M_{f}=-7$ °C, $A_{s}=13$ °C and $A_{f}=42$ °C. To observe the model’s response at different temperatures, three different operating temperatures (T) were considered for the analyses: $A_{s}<T<M_{s}$, $M_{s}<T<A_{f}$, and $T>A_{f}$. Two of these operating temperatures causes the material to exhibit the SME ($A_{s}<T<M_{s}$ and $M_{s}<T<A_{f}$) and the remaining one results in “pseudoelasticity” ($T>A_{f}$), which are the two fundamental characteristics of SMAs. These operating temperatures enabled the analysis to reveal model’s sensitivity while simulating both the shape memory and pseudoelastic properties of the material. For each of these operating temperatures, four maximum loading stresses ($\sigma_{max}$) were considered to observe the model’s response before and after full austenite-to-martensite conversion upon loading. Three of them ($\sigma_{max}$) were at different martensite volume fractions (ξ=1/3, ξ=2/3 and ξ=1) and one of them was at a higher stress when the material completes martensite transformation at ξ=1. In the simulation, the maximum loading stresses ($\sigma_{max}$) were determined by recording the values of martensite volume fraction (ξ) with the increment of stress. When ξ reached 1/3, the corresponding stress was obtained. This procedure was followed to determine the maximum loading stresses at ξ=2/3 and ξ=1. These four maximum stresses have been chosen to observe how the model behaves in terms of uncertainty and sensitivity at corresponding loading stresses of different martensite volume fractions. The temperatures and the maximum loading stresses at each temperature are presented in Table 2.

Isothermal stress–strain relationship at aforementioned temperature regions was obtained using the constitutive equations as per the Ivshin–Pence model. At these temperatures, the stress of the material is increased from 0 MPa to a maximum loading stress (as per martensite fraction volume) and, consecutively, reduced back to 0 MPa. The stress increment was selected to be 0.1 MPa. The boundary condition was such that one end of the material was kept fixed and the other end was stressed in one dimension. In all the stress–strain diagrams, the initial austenite fraction was $\alpha_{max}\left(T,0\;\mathrm{MPa}\right)$.

Two variance-based global sensitivity analysis methods were used for performing the sensitivity analysis of the Ivshin–Pence model: (1) Sobol and (2) Extended Fourier Amplitude Sensitivity Test (eFAST). Both the methods perform estimation of sensitivity measures summarizing model behavior. Sobol [67] method is based on the decomposition of a function into summands of increasing dimensions. This leads to the variance of the model output being decomposed into the variances of input parameters. Each term in the decomposition is obtained by Monte Carlo integration. The aim of Sobol sensitivity analysis is to determine how much of the variability in model output is dependent upon each of the input parameters, either upon a single parameter or upon an interaction between different parameters. The eFAST method [68] is computationally efficient than Sobol. It is based on Fourier Amplitude Sensitivity Test (FAST) [69,70]. FAST is used to compute “first order terms” while eFAST can be used to compute the “total indices”. “First order terms” refers to the “main effect” of each parameter to the variance of the output. On the other hand, “total indices” mean that interactions among the input parameters is included along with the individual contribution of each parameter to the output variance. Both Sobol and eFAST have their strength and weakness. FAST-based methods are computationally efficient over Monte Carlo, but they cost extra assumptions of smoothness as well as bias [71]. Monte Carlo-based methods show good confidence in the results when the model can be run a lot of times.

eFAST method is expected to provide better results in terms of efficiency. It is advantageous because of its robustness, especially at low sample size. This is confirmed from [68], where Saltelli et al. proposed the eFAST as a new method to perform sensitivity analysis. Convergence criteria for both models were same. Both the methods converge to the analytical values as sample size is increased. For assessing the convergence of the sensitivity index values, quantitative criteria were defined with 95% confidence interval of sensitivity index normalized from the value 0 to 1.

Both the methods to determine sensitivity indices were performed with 8 input model parameters. The analyses were performed in SobolGSA software with same user defined library developed in MATLAB as .m extension file. The outputs were calculated at same stress increment value. Both methods were performed at three operating temperatures each with four maximum stress values. The objective of performing two different sensitivity analysis is to verify the sensitivity analysis results.

The input parameters have been determined from the constitutive equations and the phase transformation equations. Equation (2) is used to calculate the strain of the material with the application of stress. The parameters $\epsilon_{m}$ and $\epsilon_{a}$ depend on $E_{a}$, $E_{m}$ and $\epsilon_{L}$ as per Equation (3). Thus, these three parameters are considered to be input parameters since they are the material constants. Equation (9) shows that the envelope function $\alpha_{max}\left(\beta \right)$ used in Equation (4) during austenite-to-martensite transformation depends on the adjustable fitting parameter $k_{1}$ and $k_{2}$. Similarly, $\alpha_{min}\left(\beta \right)$ used in Equation (5) during martensite to austenite transformation depend on $k_{3}$ and $k_{4}$ as per Equation (10). Neutrality curve β is a function of temperature, T as given in Equation (6). Hence, eight parameters are considered to be input parameters in this study: Operating temperature (*T*), elastic modulus of austenite ($E_{a}$), elastic modulus of martensite ($E_{m}$), maximum residual strain ($\epsilon_{L}$), and four adjustable fitting parameters ($k_{1}$, $k_{2}$, $k_{3}$, and $k_{4}$). These parameters are considered to be normally distributed with a coefficient of variation (COV) of 0.01. The value of COV has been such chosen to observe the output variability with small variation of input parameters. The probability distributions of the input parameters are provided in Table 3.

**Figure 1:**
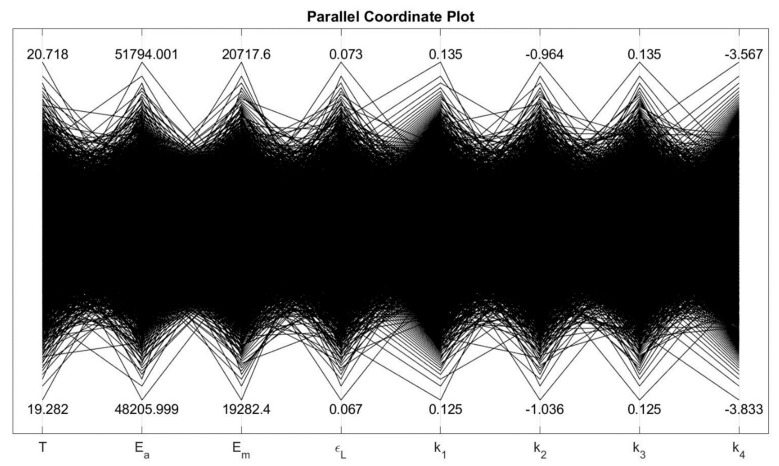
Parallel coordinate plot that shows upper and lower limits of input parameters at 20 °C.

In Table 3, the mean value denotes the deterministic value of the normally distributed input parameters and standard deviation shows the spread of the corresponding distributions. As an example of the variability introduced to the parameters, the parallel coordinate plot that presents the upper and lower limits of normally distributed input parameters at 20 °C is shown in Figure 1.

**Figure 2:**
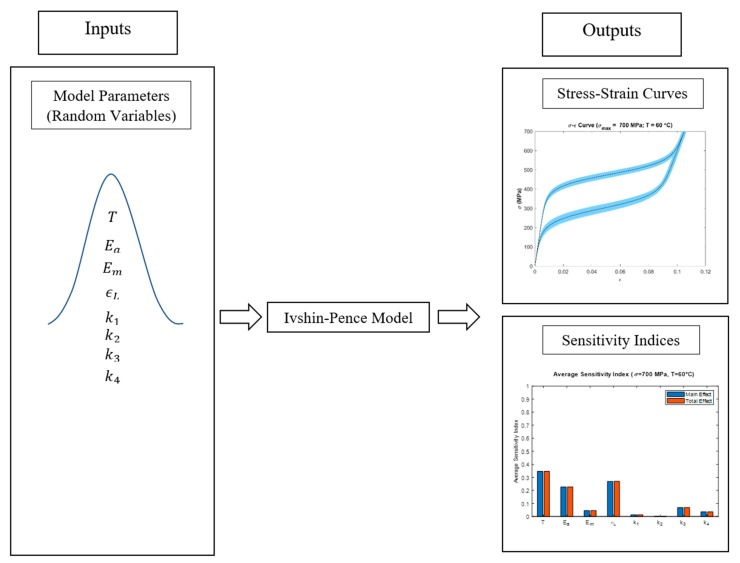
Uncertainty and sensitivity analysis outputs according to the eight input parameters.

The uncertainty analysis involves selecting random values from each of the normally distributed input parameters and calculating the output strain. The output strains were calculated for the stress value starting from 0 MPa to maximum loading stress and, consecutively, unloading back to 0 MPa. For each set of input parameter combination, a standard stress–strain relationship is obtained. The simulation was run with 3001 samples, which resulted in a band of stress–strain curves. This band is the result of the uncertainty analysis and it shows the propagation of uncertainty due to the variation in the input parameters during loading and unloading of the material. In this study, eight normally distributed parameters were used as inputs and corresponding stress–strain curves and sensitivity indices charts were generated as outputs according to the Ivshin–Pence model (Figure 2).

## 4. Results

As can be seen in Table 3, each input parameter for the model has associated uncertainty. As a result, output stress–strain curves show variability that differs depending on the simulated temperature and loading conditions. The maximum variability in strain is shown in Table 4 for each simulated operating temperature.

### 4.1. Uncertainty Analysis

**Figure 3:**
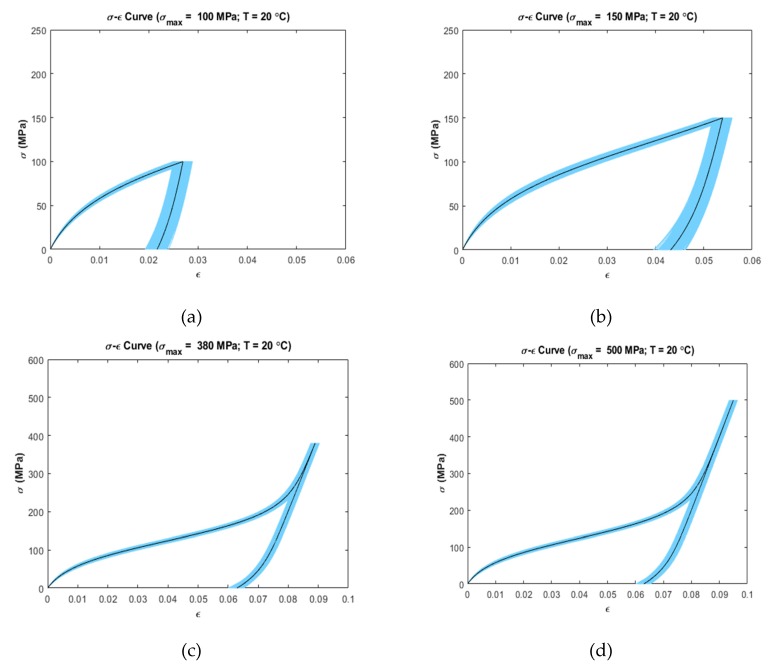
Confidence intervals (5–95 percentile) at simulated temperatures and maximum loading stress (Dark Color shows the deterministic curves); (**a**) $T=20\;{}^{\circ}C,{\;\sigma }_{\max}=100\;$ MPa (**b**) $T=20\;{}^{\circ}C,{\;\sigma }_{\max}=150$ MPa (**c**) $T=20\;{}^{\circ}C,{\;\sigma }_{\max}=380$ MPa (**d**) $T=20\;{}^{\circ}C,{\;\sigma }_{\max}=500$ MPa.

**Figure 4:**
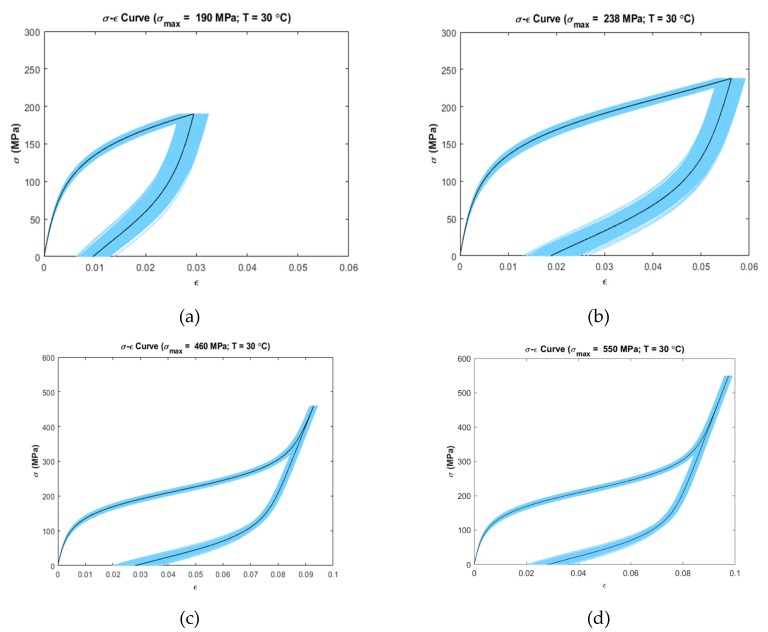
Confidence intervals (5–95 percentile) at simulated temperatures and maximum loading stress (Dark Color shows the deterministic curves); (**a**) $T=30\;{}^{\circ}C,{\;\sigma }_{\max}=190$ MPa (**b**) $T=30\;{}^{\circ}C,{\;\sigma }_{\max}=238$ MPa (**c**) $T=30\;{}^{\circ}C,{\;\sigma }_{\max}=460$ MPa (**d**) $T=30\;{}^{\circ}C,{\;\sigma }_{\max}=550$ MPa.

**Figure 5:**
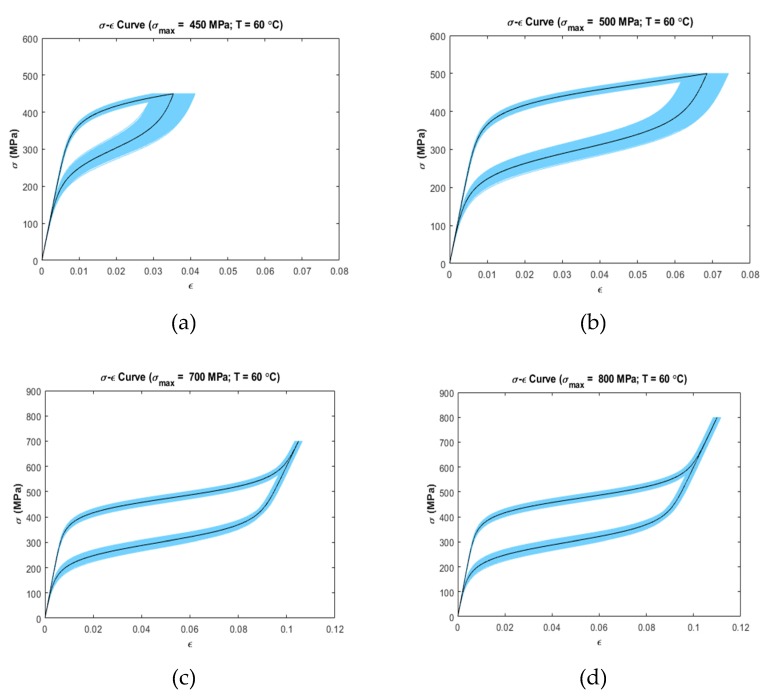
Confidence intervals (5–95 percentile) at simulated temperatures and maximum loading stress (Dark Color shows the deterministic curves): (**a**) $T=60\;{}^{\circ}C,{\;\sigma }_{\max}=450$ MPa (**b**) $T=60\;{}^{\circ}C,{\;\sigma }_{\max}=500$ MPa (**c**) $T=60\;{}^{\circ}C,{\;\sigma }_{\max}=700$ MPa (**d**) $T=60\;{}^{\circ}C,{\;\sigma }_{\max}=800$ MPa.

Uncertainty analysis was carried out by calculating 5–95% confidence intervals on the stress–strain data at several temperatures and loading conditions (Figure 3, Figure 4 and Figure 5). It was observed that the stress–strain curves generated by the model showed variation in uncertainty depending on the operating temperature and loading region.

Figure 3 shows the uncertainty propagation at 20 °C ($A_{s}<T<M_{f}$) at $\sigma_{max}=100\;\mathrm{MPa}$ (Figure 3a), $\sigma_{max}=150\;\mathrm{MPa}$ (Figure 3b), $\sigma_{max}=380\;\mathrm{MPa}$ (Figure 3c), and $\sigma_{max}=500\;\mathrm{MPa}$ (Figure 3d). In all four cases, the variability in the linear loading region is relatively low as compared to the end of the loading region. It can be observed that Figure 3a,b show increased variability in the unloading region as compared to the loading region. On the other hand, Figure 3c,d shows consistent variability at the end of loading region and in the unloading region.

Figure 4 and Figure 5 show the confidence interval curves at 30 °C ($M_{f}<T<A_{f}$) and at 60 °C ($T>A_{f}$), respectively. Both figures present a similar trend in variability, i.e., the linear loading region has low variability in strain than the non-linear loading zone. The unloading zone shows increased variability for the first two cases at every temperature simulated (Figure 4a,b and Figure 5a,b). For the remaining cases (Figure 4c,d and Figure 5c,d), the linear unloading region shows low variability than the non-linear portion of the unloading zone. For Figure 5c,d, the ending unloading region shows zero variability where the unloading curves meet with the initial loading curves.

Figure 3 and Figure 4 show uncertainty propagation in SME characteristics of SMAs and Figure 5 shows uncertainty propagation in “pseudoelastic” behavior of SMAs as per the Ivshin–Pence model.

### 4.2. Sensitivity Analysis

**Figure 6:**
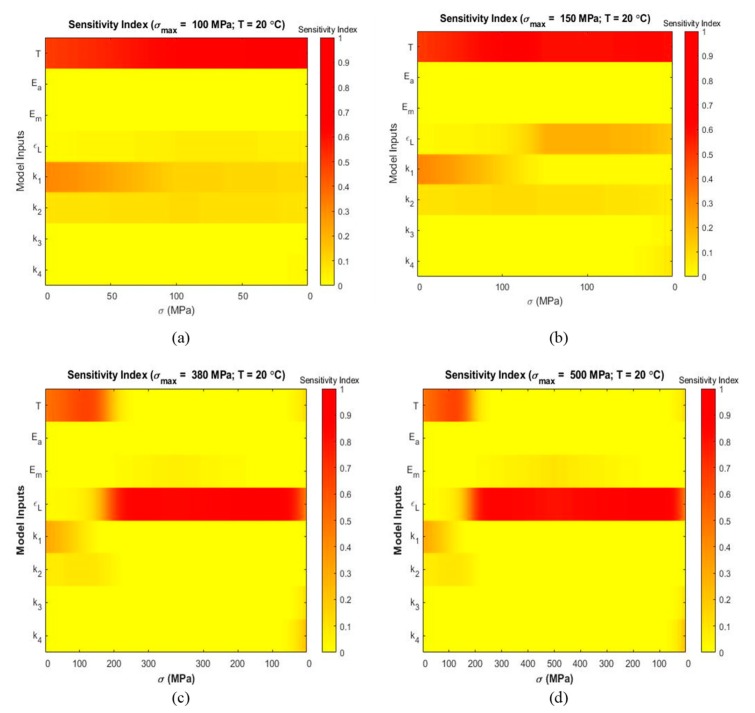
Sensitivity index distribution during loading and unloading based on eFAST sensitivity analysis at simulated temperatures (the corresponding stress values are shown in horizontal axis and the inputs are shown in vertical axis): (**a**) $T=20\;{}^{\circ}C,{\;\sigma }_{\max}=100$ MPa (**b**) $T=20\;{}^{\circ}C,{\;\sigma }_{\max}=150$ MPa (**c**) $T=20\;{}^{\circ}C,{\;\sigma }_{\max}=380$ MPa (**d**) $T=20\;{}^{\circ}C,{\;\sigma }_{\max}=500$ MPa.

**Figure 7:**
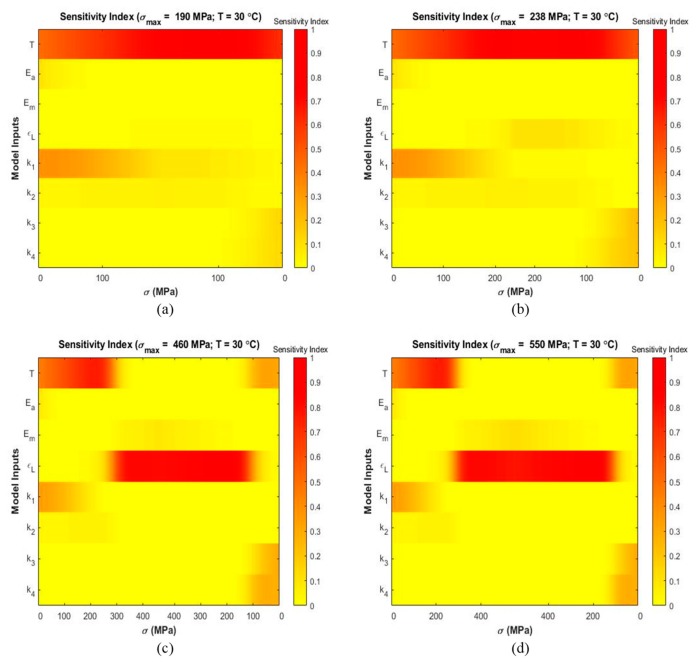
Sensitivity index distribution during loading and unloading based on eFAST sensitivity analysis at simulated temperatures (the corresponding stress values are shown in horizontal axis and the inputs are shown in vertical axis): (**a**) $T=30\;{}^{\circ}C,{\;\sigma }_{\max}=190$ MPa (**b**) $T=30\;{}^{\circ}C,{\;\sigma }_{\max}=238$ MPa (**c**) $T=30\;{}^{\circ}C,{\;\sigma }_{\max}=460$ MPa (**d**) $T=30\;{}^{\circ}C,{\;\sigma }_{\max}=550$ MPa.

**Figure 8:**
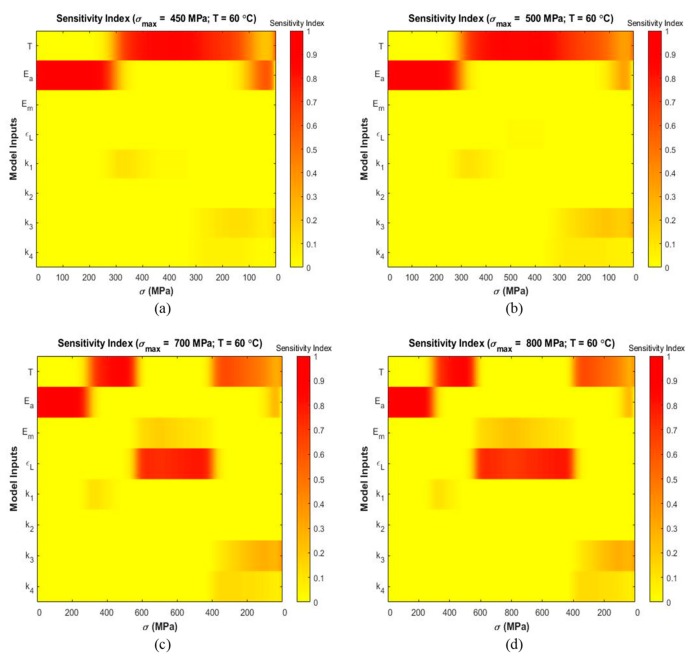
Sensitivity index distribution during loading and unloading based on eFAST sensitivity analysis at simulated temperatures (the corresponding stress values are shown in horizontal axis and the inputs are shown in vertical axis): (**a**) $T=60\;{}^{\circ}C,{\;\sigma }_{\max}=450$ MPa (**b**) $T=60\;{}^{\circ}C,{\;\sigma }_{\max}=500$ MPa (**c**) $T=60\;{}^{\circ}C,{\;\sigma }_{\max}=700$ MPa (**d**) $T=60\;{}^{\circ}C,{\;\sigma }_{\max}=800$ MPa.

Variance-based sensitivity analyses were performed to analyze the effect of each parameter on the output strain and to determine the most influential parameters at several temperatures and maximum stress levels. Figure 6, Figure 7 and Figure 8 present stress-dependent sensitivity index distribution for each parameter at four operating temperatures. At every level of stress increment, the color bar shows the sensitivity index where red denotes a sensitivity index of 1.0 and yellow denotes a sensitivity index of 0.0. The other sensitivity indices lie in between these two extremes. It is observed that the sensitivity index of a parameter varies with temperature and loading condition. For each parameter, main and total indices were also calculated using Sobol and eFAST sensitivity analysis techniques to determine the average sensitivity and total sensitivity indices. The total sensitivity indices show possible interaction between the parameters.

Figure 6 present sensitivity index distribution at 20 °C ($A_{s}<T<M_{f}$) at $\sigma_{max}=100\;\mathrm{MPa}$ (Figure 6a), $\sigma_{max}=150\;\mathrm{MPa}$ (Figure 6b), $\sigma_{max}=380\;\mathrm{MPa}$ (Figure 6c), and $\sigma_{max}=500\;\mathrm{MPa}$ (Figure 6d). It is evident from Figure 6a,b that temperature T is the significant parameter throughout the loading and unloading portion and k1 has some significance in the loading region. Figure 6c,d show that maximum residual strain $\epsilon_{L}$ has the most contribution to the overall strain variability after the initial loading region. Temperature T shows some significance during the initial loading zone.

Figure 7 shows sensitivity index distribution at 30 °C ($M_{f}<T<A_{f}$) at $\sigma_{\max}=190$ MPa (Figure 7a),${\;\sigma }_{\max}=238$ MPa (Figure 7b), $\sigma_{\max}=460$ MPa (Figure 7c), and $\sigma_{\max}=550$ MPa (Figure 7d). From Figure 7a,b it is prominent that temperature T is the most influential parameter during loading and unloading. Parameter k1 shows some significance during the loading zone. Figure 7c,d presents that $\epsilon_{L}$ is the most and T is the second most significant parameter.

The material shows “pseudoelasticity” at temperatures $T>A_{f}$. Figure 8 depicts the sensitivity index distribution of the material while displaying “pseudoelastic” behavior at 60 °C at $\sigma_{\max}=450$ MPa (Figure 8a), $\sigma_{\max}=500$ MPa (Figure 8b), $\sigma_{\max}=700$ MPa (Figure 8c), and $\sigma_{\max}=800$ MPa (Figure 8d). Figure 8a,b show that the model is sensitive to the austenite modulus $E_{a}$ in the initial loading zone. It shows sensitivity to T from 300 MPa of the loading zone and continues up to 100 MPa of the unloading zone. It is observed from Figure 8c,d that $\epsilon_{L}$, T and $E_{a}$ are the significant parameters.

**Figure 9:**
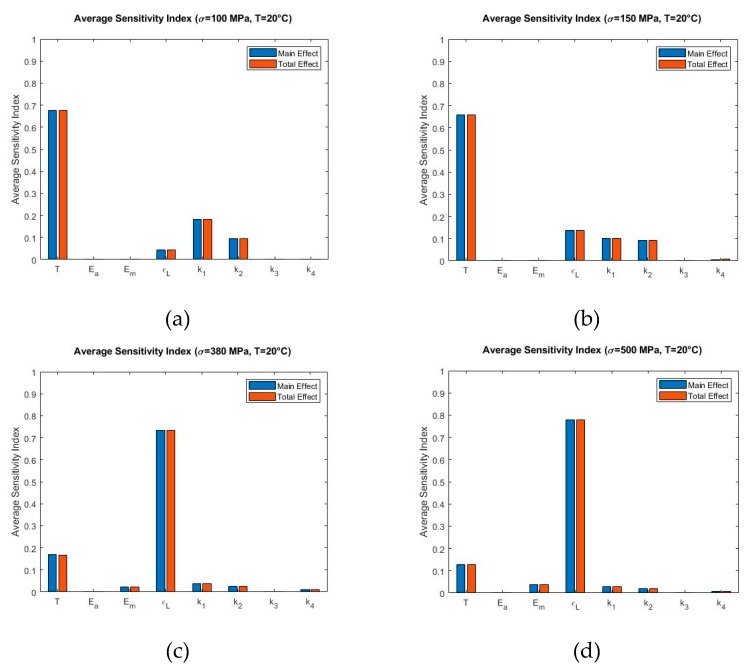
Sobol average sensitivity indices at simulated temperatures and maximum loading: (**a**) $T=20\;{}^{\circ}C,{\;\sigma }_{\max}=100$ MPa (**b**) $T=20\;{}^{\circ}C,{\;\sigma }_{\max}=150$ MPa (**c**) $T=20\;{}^{\circ}C,{\;\sigma }_{\max}=380$ MPa (**d**) $T=20\;{}^{\circ}C,{\;\sigma }_{\max}=500$ MPa.

**Figure 10:**
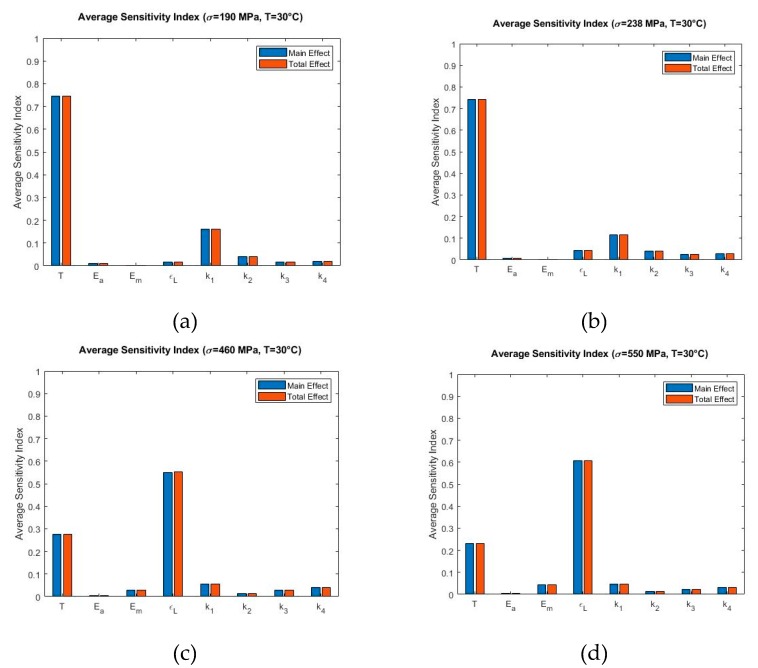
Sobol average sensitivity indices at simulated temperatures and maximum loading: (**a**) $T=30\;{}^{\circ}C,{\;\sigma }_{\max}=190$ MPa (**b**) $T=30\;{}^{\circ}C,{\;\sigma }_{\max}=238$ MPa (**c**) $T=30\;{}^{\circ}C,{\;\sigma }_{\max}=460$ MPa (**d**) $T=30\;{}^{\circ}C,{\;\sigma }_{\max}=550$ MPa.

**Figure 11:**
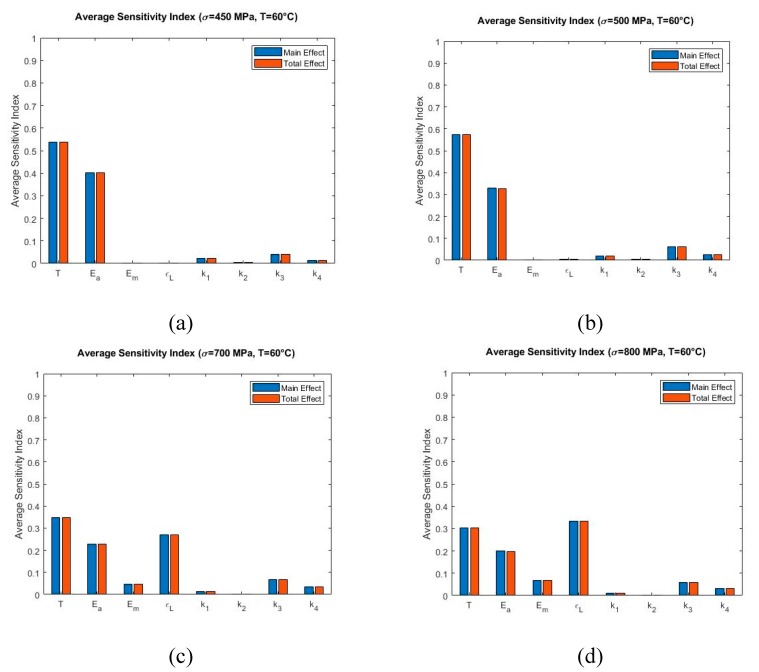
Sobol average sensitivity indices at simulated temperatures and maximum loading stress: (**a**) $T=60\;{}^{\circ}C,{\;\sigma }_{\max}=450$ MPa (**b**) $T=60\;{}^{\circ}C,{\;\sigma }_{\max}=500$ MPa (**c**) $T=60\;{}^{\circ}C,{\;\sigma }_{\max}=700$ MPa (**d**) $T=60\;{}^{\circ}C,{\;\sigma }_{\max}=800$ MPa.

Figure 9, Figure 10 and Figure 11 show the Sobol sensitivity analysis results of each parameter at different temperatures in terms of average sensitivity indices. The main effect and total effect sensitivity indices at each stress increment and decrement level were used to obtain the average main effect and average total effect sensitivity indices. Specifically, Figure 9 shows the Sobol sensitivity analysis of the model at 20 °C at $\sigma_{max}=100\;\mathrm{MPa}$ (Figure 9a), $\sigma_{max}=150\;\mathrm{MPa}$ (Figure 9b), $\sigma_{max}=380\;\mathrm{MPa}$ (Figure 9c), and $\sigma_{max}=500\;\mathrm{MPa}$ (Figure 9d). Figure 9a,b present that T has the highest sensitivity index. At increased max stress level, the contribution of significance shifts from T to $\epsilon_{L}$ (Figure 9c,d). This trend continues at temperature 30 °C in all simulated stress levels where $\sigma_{\max}=190$ MPa (Figure 10a), $\sigma_{\max}=238$ MPa (Figure 10b), $\sigma_{\max}=460$ MPa (Figure 10c), and $\sigma_{\max}=550$ MPa (Figure 10d). The only exception is that T is not influential at higher stress levels (Figure 9c,d), but it shows some significance in Figure 10c,d.

The Sobol sensitivity analysis results at 60 °C are shown in Figure 11 where $\sigma_{\max}=450$ MPa (Figure 11a), $\sigma_{\max}=500$ MPa (Figure 11b), $\sigma_{\max}=700$ MPa (Figure 11c), and $\sigma_{\max}=800$ MPa (Figure 11d). From Figure 11a,b it is observed that Temperature T is the most significant parameter and austenite modulus $E_{a}$ is the second most influential parameter. At higher stress values, the model tends to be sensitive to $\epsilon_{L}$ and its contribution increases while the contribution of T and $E_{a}$ decrease (Figure 11c,d).

For all cases, there is no significant interaction among the parameters as the main effect and total effect are in close agreement with each other. The parameters $E_{m}$, $k_{3}$ and $k_{4}$ are not influential in any of these cases. The Sobol sensitivity analysis results have been verified using eFAST sensitivity analysis. The results from both methods are in good agreement, which verifies the results of the Sobol analysis.

## 5. Discussion

In this study, the one-dimensional Ivshin–Pence SMA constitutive model was analyzed to demonstrate the effect of input parameter variability to the model’s sensitivity and output stress–strain relationship at various simulated operating temperature ranges and loading conditions. A probabilistic approach by assigning normally distributed probability density function to the model’s input parameters was employed. The sensitivity analysis provides useful insights to identify the most influential parameters of a model and the uncertainty analysis shows how the uncertainty in the input parameters propagates to the output via the constitutive equations. The results presented in this paper can be beneficial in designing engineering applications or experiments at particular temperature ranges and loading conditions. In the following paragraphs, we discuss the results from our analysis and provide recommendation for proper use of the Ivshin–Pence model in designing SMA-based applications.

### 5.1. Uncertainty Analysis

At 20 °C ($A_{s}<T<M_{f}$) with maximum stress 100 MPa, 150 MPa, 380 MPa and 500 MPa (Figure 3a–d), the linear loading region shows very low variability as compared to the rest of the loading region. Initially in this region, the austenite fraction of the material is $\alpha_{\max}\left(20,0\right)$ which results in α=0.96, i.e., the material is mostly austenite. The neutrality curve β in Equation (6) has a quadratic component of stress, σ. Therefore, lower value of stress in this region results in lower decrement of β. With the uncertainty present in the adjustable fitting parameters ($k_{1},\;k_{2}$) as per Equation (9), the low decrement rate of β does not contribute significantly in decreasing α. Consequently, low variability is present in the output strain. The non-linear loading zone shows increased variability. This is because of the fact that increased stress, σ causes the individual phase strains to incur variability due to the uncertainty in the elastic modulus of austenite ($E_{a}$), elastic modulus of martensite ($E_{m}$), and maximum residual strain ($\epsilon_{L}$) as demonstrated by Equation (3). At the same temperature, for maximum stress of 100 MPa and 150 MPa (Figure 3a,b), the material does not fully convert from austenite to martensite. The non-linear unloading zone shows increased variability in comparison with the non-linear loading zone. For maximum stress of 380 MPa and 500 MPa (Figure 3c,d), however, it is observed that constant variability is present at the end of loading region and in the linear unloading region. This occurs mainly because, when the transformation from austenite to martensite completes, the austenite fraction becomes zero. This causes the austenite-phase strain to become zero in calculating strain as per Equation (3), i.e., the parameters $E_{a}$, $k_{3}$, $k_{4}$,T are not involved in the constitutive equation resulting in low variability in the linear unloading region.

At 30 °C ($M_{s}<T<A_{f}$) with maximum stress of 190 MPa, 238 MPa, 460 MPa, and 550 MPa (Figure 4a–d), the linear loading region has low variability in strain than the non-linear loading zone. This is the same for 60 °C temperature ($T>A_{f}$) with all the simulated cases including maximum stress of 450 MPa, 500 MPa, 700 MPa, and 800 MPa (Figure 5a–d). In both temperature zones, the material initially is in austenite phase (α=1). This causes the contribution of individual phase strain of martensite, $\epsilon_{m}$ to become zero as per Equation (3). Thus, the terms $E_{m}$, $k_{1}$, $k_{2}$, T and $\epsilon_{L}$ are not involved in the constitutive equation, which results in low variability in the initial loading zone. On the other hand, in the non-linear loading zone, the material undergoes phase transformation from austenite to martensite. As a result, the parameters $E_{a}$, $E_{m}$, $k_{1}$, $k_{2}$, T and $\epsilon_{L}$ come into effect in the constitutive equation. Therefore, higher variability is prominent in this region. At 30 °C with maximum stress of 190 MPa and 238 MPa (Figure 4a,b) and at 60 °C with maximum stress of 450 MPa and 500 MPa (Figure 5a,b), the non-linear unloading zone shows increased variability than the non-linear loading zone. At these maximum stresses, the material is in a mixed phase consisting of austenite and martensite. Unloading from this stress value involves the parameters $E_{a}$, $E_{m}$, $k_{3}$, $k_{4}$, T and $\epsilon_{L}$ to come into effect in the constitutive equation. The unloading includes the martensite to austenite-phase transformation kinetics. Moreover, the parameter $k_{4}$ (fitting parameter during martensite to austenite transformation) has a higher spread than $k_{2}$ (fitting parameter during austenite-to-martensite transformation) which causes the increased uncertainty inherent in $k_{4}$ to propagate to the output. As a result, high variability is observed in the output strain for these cases. On the other hand, at 30 °C with maximum stress of 460 MPa and 550 MPa (Figure 4c,d) and at 60 °C with maximum stress of 700 MPa and 800 MPa (Figure 5c,d), the initial linear unloading zone shows low variability than the non-linear portion of unloading zone. At these maximum stresses, the material fully converts from austenite to martensite. As a result, when unloading, the austenite fraction is zero (α=0). Due to this, the individual austenite-phase strain becomes zero. So the parameters, $E_{a}$, $k_{3}$, $k_{4}$ and T are not involved in the constitutive equation resulting in low variability in the initial linear unloading zone. During the non-linear unloading zone, transformation from martensite to austenite takes place. Because of this, both austenite and martensite phase strains contribute to the total strain involving the parameters $E_{a}$, $E_{m}$, $k_{3}$, $k_{4}$, T and $\epsilon_{L}$. Therefore, the variability increases in this region.

For 60 °C temperature with maximum stress of 700 MPa and 800 MPa (Figure 5c,d), last portion of the linear unloading zone shows zero variability due to the fact that martensite to austenite conversion is fully complete at this zone (α=1). This results in making the contribution of martensite phase strain to become zero. Therefore, $E_{m}$, $k_{3}$, $k_{4}$, T, and $\epsilon_{L}$ are not involved in the transformation kinetics resulting in zero variability in this zone.

It was observed that the operating temperature has a significant effect on the output variability. With increasing temperature, the general tendency is that the strain variability increases. This is because of the temperature range in which the phase transformation occurs. For example, in the temperature range $T<A_{f}$, the variability is lower because the material cannot undergo the complete reverse transformation from martensite to austenite. On the contrary, when $T>A_{f}$, the variability increases as in this temperature zone, the material can transform fully from martensite to austenite during unloading.

### 5.2. Sensitivity Analysis

The sensitivity index distribution reveals that at 20 °C with maximum stress of 100 MPa and 150 MPa (Figure 6a,b), the model is sensitive to the operating temperature T throughout the loading and unloading region. Fitting parameter $k_{1}$ shows some contribution in terms of sensitivity index during the loading zone. Maximum residual strain $\epsilon_{L}$ shows some significance during the unloading zone for the case when maximum stress is 150 MPa (Figure 6b). Transformation from austenite to martensite and vice versa is governed by envelope functions $\alpha_{\max}(\beta$) and $\alpha_{\min}(\beta$), where β is a function of temperature T and stress σ. Therefore, for Figure 6a,b, the model is mostly sensitive to temperature T. The significance of $k_{1}$ is because during loading, transformation from austenite to martensite involves the parameter $k_{1}$. The total strain and residual strain due to the application of stress is less in comparison with the other cases (at maximum stress of 380 MPa and 500 MPa). Thus, low significance of maximum residual strain ($\epsilon_{L}$) is anticipated. This is also depicted in the Sobol average sensitivity index in Figure 9a,b, which shows that T is the most significant parameter. At the same temperature, with maximum stress of 380 MPa and 500 MPa, the model is sensitive to the parameter T during the linear loading zone. The maximum residual strain ($\epsilon_{L}$) becomes dominant in the remaining loading and unloading portion of the sensitivity index distribution graph (Figure 6c,d). At higher maximum stress conditions (Figure 6c,d), the total and residual strain of the material tend to increase, which explains the significance of $\epsilon_{L}$.

Sensitivity analysis performed at 30 °C with maximum stress of 190 MPa and 238 MPa shows that temperature T is dominant in the entire loading and unloading region (Figure 7a,b). The fitting parameter $k_{1}$ contributed to the output variability especially during the loading region. As mentioned previously, the envelope functions $\alpha_{\max}(\beta$) and $\alpha_{\min}(\beta$) are employed during the phase transformations. The neutrality curve β depends on temperature T and stress σ as per Equation (6). Therefore, at maximum stress 190 MPa and 238 MPa, the model is sensitive to temperature T. Sobol average sensitivity index in Figure 10a,b show that temperature is the most significant parameter. The envelope function reveals that transformation from austenite to martensite involves the parameter $k_{1}$. Therefore, contribution of $k_{1}$ is expected. At higher maximum stress conditions (460 MPa and 550 MPa) as in Figure 7c,d, the model exhibits sensitivity to temperature until stress level of 300 MPa during loading. Then, it shows sensitivity to $\epsilon_{L}$ during the rest of the loading zone and up to the initial loading zone during unloading. At the end of the loading, the material converts fully from austenite to martensite. As a result, the austenite fraction becomes zero for which the total strain is only dependent on the martensite phase strain which involves the term $\epsilon_{L}$. Additionally, during the austenite-to-martensite transformation, contribution of martensite phase strain increases to the total strain. Therefore, significance of $\epsilon_{L}$ is observed. Figure 10c,d show that $\epsilon_{L}$ and T have the highest Sobol sensitivity indices among all parameters.

At 60 °C with maximum stress of 450 MPa and 500 MPa (Figure 8a,b), the model is most sensitive to the parameter $E_{a}$ in the initial loading zone. Thereafter, it exhibits sensitivity to T for all remaining regions. No other parameter shows significant contribution at these maximum stress conditions. The material at this temperature initially is in austenite phase (α=1) and the total strain is only dependent on the austenite-phase strain. Therefore, contribution of the austenite elastic modulus $E_{a}$ with the increment of stress in expected as per Equation (3). Figure 11a,b present that temperature T and $E_{a}$ have the highest Sobol sensitivity indices. At higher maximum stress levels of 700 MPa and 800 MPa (Figure 8c,d), the parameter $E_{a}$ shows contribution during the linear initial loading region followed by the contribution of T upto the ending of the non-linear loading region. The span of significance of $\epsilon_{L}$ starts next up to the ending of the linear unloading region. Initially the material is in the austenite phase for which $E_{a}$ becomes dominant. When the material undergoes phase transformation and reaches close to austenite fraction of zero (α=0), the total strain becomes dependent on martensite phase strain. Thus, the significance of $\epsilon_{L}$ is prominent. From Figure 11c,d, it can be observed that the $\epsilon_{L}$ is the first, T is the second and $E_{a}$ is the third influential parameter in terms of Sobol average sensitivity indices.

As per the sensitivity analyses, it is observed that the “main effect” is similar to the “total effect”. This is because the parameters are not interacting with one another. It is dependent on the model and how the parameters are being used in the model. Total effect is significant in sensitivity analysis. If it is zero for a variable, it means neither the variable nor its interactions have any influence. Therefore, total sensitivity index can be used to identify the essential variables [72].

For engineering application designers and analysts, it is a fundamental task to perform material characterization which consists of ability to predict response of some application and careful planning and execution for correct model calibration. When the Ivshin–Pence model is used in design engineering, proper attention should be given in incorporating uncertainty into the input parameters when operating temperature is $T>A_{f}$. Because from the variability data presented in Table 4, it is observed that variability increases with increasing temperature which suggests uncertainty propagation is high in that temperature range. Additionally, the material shows increased variability in the unloading zone when operating at the loading conditions where austenite-to-martensite transformation is not fully complete. Depending on the material and type of application, this behavior may lead to failure if the uncertainty in the output is not considered during design procedure. Therefore, it is recommended to use the results of this analysis into account during unloading at martensite fraction ξ<1.

Finally, the most influential parameters of the Ivshin–Pence model are listed in Table 5. From the table, it can be observed that the model is sensitive to certain model parameters at different operating temperature conditions, i.e., in other words, for temperature ranges when the model shows SME and pseudoelastic effect. This clearly indicates the necessity of this study in using the SMA’s in engineering applications or research initiatives as real-life applications are performed at varying temperature and loading conditions. Therefore, proper knowledge and understanding on the parameters for which the model is most sensitive can help better design applications eliminating the risk of failure due to the uncertainty in the input parameters.

## 6. Conclusions

In this paper, sensitivity and uncertainty analysis were presented for the Ivshin–Pence shape memory alloy constitutive model. The model involves parameters which are subjected to uncertainty and random variability. The uncertainty propagates to the output causing variability in the output stress–strain relationship at different operating temperatures and loading conditions. Two widely recognized sensitivity analysis approaches, Sobol and eFAST, were performed to determine the sensitivity indices of each parameter. These indices provide a clear idea about the parameters for which the model is most sensitive. The uncertainty analysis shows the trend of variability in the output caused by the uncertainty present in the input parameters. It also suggests that any variability present in the parameters can significantly impact the model output for which the material may be susceptible to failure. The results in this work can be used for creating simulations which represents material behavior in engineering or commercial applications. Future work can include other SMA material to perform sensitivity and uncertainty analysis of the Ivshin–Pence model or any other SMA model at different operating temperatures and loading conditions.

## Figures and Tables

**Table 1:** Material Properties Ivshin et al. [58].

Parameter	Description	Deterministic Value	Unit
T	Temperature	20, 30, 60	°C
$E_{a}$	Elastic modulus of Austenite	50,000	MPa
$E_{m}$	Elastic modulus of Martensite	20,000	MPa
$\epsilon_{L}$	Maximum residual strain	0.07	N/A
$M_{s}$	Martensite Start Temperature	22.00	°C
$M_{f}$	Martensite Finish Temperature	−7.00	°C
$A_{s}$	Austenite Start Temperature	13.00	°C
$A_{f}$	Austenite Finish Temperature	42.00	°C
$k_{1}$	Adjustable Fitting Parameter #1	0.13	/°C
$k_{2}$	Adjustable Fitting Parameter #2	−1.00	N/A
$k_{3}$	Adjustable Fitting Parameter #3	0.13	/°C
$k_{4}$	Adjustable Fitting Parameter #4	−3.70	N/A

**Table 2:** Simulated Operating Temperature and Loading Conditions.

Temperature (°C)	Martensite Volume Fraction, ξ	$\mathrm{Maximum}\;\mathrm{Loading}\;\mathrm{Stress},\;\sigma_{max}$	Region
20	1/3	100	$A_{s}<T<M_{s}$
	2/3	150	
	1	380	
	1	500	
30	1/3	190	$M_{s}<T<A_{f}$
	2/3	238	
	1	460	
	1	550	
60	1/3	450	$T>A_{f}$
	2/3	500	
	1	700	
	1	800	

**Table 3:** Probability distribution of input parameters.

Parameter	Distribution	Mean Value	Standard Deviation	Unit
T	Normal	20, 30, 60	0.20, 0.30, 0.60	°C
$E_{a}$	Normal	50,000	500	MPa
$E_{m}$	Normal	20,000	200	MPa
$\epsilon_{L}$	Normal	0.07	0.0007	N/A
$k_{1}$	Normal	0.13	0.0013	/°C
$k_{2}$	Normal	−1.00	0.01	N/A
$k_{3}$	Normal	0.13	0.0013	/°C
$k_{4}$	Normal	−3.70	0.037	N/A

**Table 4:** Maximum Variability in strain.

Operating Temperature *T*(°C)	Maximum Variability
20	15–18%
30	38–48%
60	53–168%

**Table 5:** Most influential parameters of the Ivshin–Pence model.

Temperature, T(°C)	Most Significant Model Parameter	SMA Behavior
20 ($A_{s}<T<M_{f}$)	T, $\epsilon_{L}$, $k_{1}$	Shape Memory Effect
30 ($M_{s}<T<A_{f}$)	T, $\epsilon_{L}$, $k_{1}$	
60 ($T>A_{f})$	T, $E_{a}$, $\epsilon_{L}$	Pseudoelastic Effect
